# Medical cannabis regulation: an overview of models around the world with emphasis on the Brazilian scenario

**DOI:** 10.1186/s42238-022-00142-z

**Published:** 2022-06-16

**Authors:** Maíra Ribeiro de Souza, Amélia Teresinha Henriques, Renata Pereira Limberger

**Affiliations:** 1 Laboratório de Análises e Pesquisas em Toxicologia, Faculdade de Farmácia, Universidade Federal do Rio Grande do Sul (UFRGS), Porto Alegre, RS 90610-000 Brazil; 2 Laboratório de Farmacognosia e Controle da Qualidade de Fitoterápicos, Faculdade de Farmácia, Universidade Federal do Rio Grande do Sul (UFRGS), Porto Alegre, RS 90610-000 Brazil; 3 Agência Nacional de Vigilância Sanitária (ANVISA), Brasília, DF 71205-050 Brazil

**Keywords:** Medical cannabis, Regulatory framework, Patient access, Brazil

## Abstract

*Cannabis sativa* has accompanied humankind since ancient times, permeating the most diverse aspects of its existence, among which the search for health promotion and well-being stands out. Nevertheless, during the twentieth century, a series of restrictions and controls have been adopted internationally to prevent the abusive use of this species. Despite that, there has been an increased demand for the medical use of cannabis and its derivatives in the last few decades, especially among patients with debilitating conditions for which the existing therapeutic alternatives are limited. Accordingly, several countries have adopted regulatory strategies to allow access to cannabis-based products. This study aimed to overview the existing regulatory frameworks for medical cannabis around the world, focusing on the current Brazilian scenario. In addition to supply and access regulation aspects, some quality-related issues regarding cannabis-based pharmaceutical products were addressed, with emphasis on risks to patients. The literature research was performed between October 2020 and March 2021. According to the retrieved information, by the time the data collection was completed, thirty-six countries had already implemented regulatory frameworks regarding medical cannabis, and sixteen countries had models under development or in the process of implementation. The characteristics of the assessed regulatory strategies vary considerably from country to country, reflecting sociocultural, historical, and political aspects. Among the key aspects that differed between the assessed models, one can highlight the type of cannabis products that are made available and the technical requirements applied to them, as well as the possible access mechanisms. Different supply regulation strategies were also observed regarding cannabis cultivation, production licensing, and distribution mechanisms. In Brazil, an evolution of the regulatory framework has been noticeable since 2015, even though pending points are still to be addressed, among which are the species’ cultivation and the access to it for scientific research purposes. Constructing a regulatory model which provides access to good quality cannabis-based medicines that may meet the patient’s needs is still a challenge in the coming years, requiring the engagement of various stakeholders, including regulators, members of the academic community, prescribing professionals, and patients.

**Supplementary Information:**

The online version contains supplementary material available at 10.1186/s42238-022-00142-z.

## Background

The species *Cannabis sativa* L. (Cannabaceae) has been cultivated by humankind since the emergence of the first agricultural civilizations, being adapted to diverse uses, including as a source of food, oil, and fiber, as well as for medicinal, recreational, and religious purposes (Robert Clarke 2013; Bonini et al. 2018; Pisanti and Bifulco 2017; Koltai and Namdar 2020; Seddon and Floodgate 2020). This human-driven selection process over several centuries is the origin of the phenotypic diversity of species that remains nowadays (Bonini et al. 2018; Hillig 2004).

Although there are records of the medicinal use of the species in various cultures since ancient times, it was not until the nineteenth century that the interest in exploring its therapeutic potential became outstanding in the Occident (Robert Clarke 2013; Seddon and Floodgate 2020). This period was marked by the widespread use of commercial cannabis-based products, which were mainly used as anti-inflammatory, analgesic, anti-emetic, and anti-convulsant (Bonini et al. 2018; Pisanti and Bifulco 2017). This was reflected in the inclusion of monographs of the species in some official compendia, including the United States Pharmacopeia (1850) (Madras 2015; Giancaspro et al. 2016), the British Pharmacopoeia (1888) (Giancaspro et al. 2016), and the 1st edition of Brazilian Pharmacopoeia (1929), which described a raw material used to obtain Indian hemp extracts (Rocha et al. 2020).

Nevertheless, the active constituents of the species were not known at that time and there was no adequate standardization of the commercial pharmaceutical products, which caused their therapeutic effects to be highly variable. In addition, therapeutic alternatives of synthetic origin emerged at the beginning of the twentieth century. Together, these factors lead to a dramatic decline in interest in the medical use of this species (Pisanti and Bifulco 2017; Madras 2015; Kalant and Porath-waller 2016). Despite this, there were no reports of serious cases of intoxication by cannabis products (Pisanti and Bifulco 2017).

Also during that period, observation of potential risks associated with cannabis use, including the abuse and chemical dependency, has raised concerns from government authorities in several countries, leading to the formulation of restrictive laws regarding the growth, commercialization, and consumption of the species (Pisanti and Bifulco 2017; Seddon and Floodgate 2020; Madras 2015; European Monitoring Centre for Drugs and Drug Addiction 2018a). In Brazil, trade-in all substances considered to be narcotics was prohibited in 1921 and, in 1932, Decree 20.9319 turned out to criminalize the user, establishing drug addiction as a disease subject to compulsory hospitalization (Oliveira 2017). Restrictive policies have been intensified after the United Nations conventions on the control of narcotic drugs (1961, 1971, and 1988), given the classification of cannabis and cannabis resin as schedule I and IV substances, which represents the heaviest control regime of the 1961 Convention, reserved for particularly harmful substances (Pisanti and Bifulco 2017; Madras 2015; Santé Canada 2016; World Health Organization 2014; United Nations 1973).

Despite that, cannabis remained the most widely used illicit drug in the world (Santé Canada 2016; World Health Organization 2018). Accordingly, numerous government authorities have recognized not only the failures of the current repression policies to control the abusive use of the species but also their negative impacts, including the worsening of social injustices and ineffective spending of public resources (Seddon and Floodgate 2020; Santé Canada 2016; Mackay and Phillips 2016). In this context, there is a growing tendency to discuss cannabis control as a public health issue in international dialogs (Santé Canada 2016; World Health Organization 2018; Mackay and Phillips 2016), among which stands out the Extraordinary Session of the 2016 United Nations General Assembly on the World Drug Problem, when the possibility of reviewing the repression policies defined in the abovementioned conventions was signaled, with manifestations favorable to the expansion of the autonomy of the signatory countries (Mackay and Phillips 2016). Among the guidelines defined in the 2016 UN Assembly, one can highlight the facilitation of access to controlled substances for medical and research purposes (Mackay and Phillips 2016).

In spite of the limitations imposed by the aforementioned prohibitionist policies, the research into the phytochemical composition and the pharmacological properties of *C. sativa* has markedly evolved throughout the twentieth century (Pisanti and Bifulco 2017; Kalant and Porath-waller 2016; Mackay and Phillips 2016; Stockings et al. 2018). A number of non-clinical and clinical studies have been carried out in the last decades, providing scientific evidence of the effectiveness of *C. sativa* and/or its major cannabinoids in the treatment of some pathological conditions. In this regard, one can highlight the use of cannabidiol (CBD) to reduce seizures in patients with refractory epilepsy when added to conventional anti-epileptic drugs, especially in children with Dravet and Lennox-Gastaut syndromes (Stockings et al. 2018; European Medicines Agency 2019; Devinsky et al. 2017; Berkovic 2017); as well as on the use of an association of tetrahydrocannabinol (Δ^9^-THC) and CBD to reduce multiple sclerosis-related spasticity and neuropathic pain (Whiting et al. 2015; Kowal et al. 2016). There is also weak evidence of the efficacy of Δ^9^-THC and analogs for the relief of nausea and vomiting related to chemotherapy and appetite stimulation in HIV-positive patients (Whiting et al. 2015; European Monitoring Centre for Drugs and Drug Addiction 2018b; Chow et al. 2020).

Despite the pharmacological plausibility and the existence of clinical reports of the use of cannabis-based products for several other pathological conditions, including neurological and psychological disorders, such as anxiety, post-traumatic stress disorder, attention deficit hyperactivity disorder (ADHD), and depression; neurodegenerative disorders, such as amyotrophic lateral sclerosis (ALS), Parkinson’s disease, and Alzheimer’s disease; inflammatory bowel disorders; and palliative care of cancer patients, the clinical studies regarding these therapeutic claims are mostly of low quality or even absent (Whiting et al. 2015; Legare et al. 2022; Alves et al. 2020; European Monitoring Centre for Drugs and Drug Addiction (EMCDDA), 2018; Black et al. 2019). Therefore, additional clinical trials of good methodological quality are still needed in order to support the aforementioned therapeutic claims (European Monitoring Centre for Drugs and Drug Addiction 2018b; Health Products Regulatory Authority 2017; Dinis-Oliveira 2019; Russo 2016).

Regarding the safety profile of cannabis-based medicinal products, the risks associated with the short-term use have been considered analogous to those of other medicines available on the market, with serious adverse effects rarely reported in clinical trials. On the other hand, long-term risks are less known; therefore, further studies are needed to generate sufficient evidence (European Monitoring Centre for Drugs and Drug Addiction 2018b; Health Products Regulatory Authority 2017). This concern is especially relevant when it comes to the long-term use of ∆^9^-THC-containing products, especially in children and adolescents, who are more vulnerable to the deleterious effects of this cannabinoid (Health Products Regulatory Authority 2017; Crippa et al. 2016).

The aforementioned advances in knowledge of cannabis’ therapeutic properties have met interests in the economic exploitation of the potential of cannabis-based medical products, motivating debates about the revision of the previously imposed legal restrictions (Pisanti and Bifulco 2017; Kalant and Porath-waller 2016; Mackay and Phillips 2016; Kowal et al. 2016). In 2019, the World Health Organization (WHO)’s Expert Committee on Drug Dependence (ECDD) recommended the removal of cannabis and cannabis resin from schedule IV of the 1961 UN convention, while maintaining them in schedule I (Expert Committee on Drug Dependence 2019). In December 2020, the United Nations Commission on Narcotic Drugs (CND) decided to follow this ECDD’s recommendation (United Nations Commission on Narcotic Drugs 2020). This decision symbolizes the recognition by the UN of the cannabis’ potential for therapeutic use.

Notwithstanding the recent cannabis rescheduling, the development of scientific-based regulations that address health risks related to the use of cannabis products, as well as adequate quality specifications, is still a challenge. Some of the main aspects of possible regulatory approaches that may have an impact on patients’ access to safe and effective cannabis-based medicinal products will be addressed in this review.

## Methods

Literature research was performed in Google Scholar and Science Direct using the entry terms “medical cannabis,” “regulatory framework,” and “marketing authorization,” covering information published until March 2021. Additional information was also retrieved from websites of regulatory agencies from assessed countries and gray literature relevant to the addressed topic.

A set of relevant aspects for the evaluation and comparison of the addressed regulatory models was defined, including four main subjects: (I) supply regulation, (II) demand regulation, (III) type of available products, and (IV) technical criteria related to the available products. Each of those subjects was divided into two or more subtopics treated separately. A set of categories was defined for each subtopic in order to allow a standardized classification of the evaluated models (see Supplementary Material). Figures (charts and map) were created from processed data using MS Excel®.

## Medical cannabis regulation around the world

International drug control treaties in force do not prevent signatory nations from allowing the use of cannabis for medical and scientific purposes within their territories (European Monitoring Centre for Drugs and Drug Addiction 2018a; World Health Organization 2014; European Monitoring Centre for Drugs and Drug Addiction 2018b; United Nations Office on Drugs and Crime 2013). For that, however, a set of stringent control measures are required, among which stands out the need to establish government agencies responsible for controlling the medical cannabis supply chain, which must report to the International Narcotics Control Board (INCB) (World Health Organization 2014; European Monitoring Centre for Drugs and Drug Addiction 2018b; Health Products Regulatory Authority 2017; Aguilar et al. 2018). National cannabis agencies also have the exclusive right to maintain stocks of harvested plant material, being responsible for its distribution on a wholesale scale (Health Products Regulatory Authority 2017). Furthermore, cannabis-based products should be dispensed upon prescription and used under medical supervision, based on evidence of their safety, effectiveness, and quality (European Monitoring Centre for Drugs and Drug Addiction 2018b).

The INCB considers that the cultivation of cannabis for personal medical use does not meet the minimum criteria related to the control requirements of the 1961 Convention. Therefore, signatory countries whose regulatory framework allows cannabis self-cultivation would be in breach of this treaty (Health Products Regulatory Authority 2017). Cannabis cultivation exclusively for industrial obtaining of seeds and fibers is exempted from the controls of the aforementioned treaty (United Nations Office on Drugs and Crime 2013).

It is worth mentioning that the 1961 UN Convention does not establish distinctive restrictive control regimes for different cannabis variants, regardless of their variety concerning cannabinoid profiles, which is known to be decisive for the psychopharmacological properties of the species (World Health Organization 2014). On the other hand, cannabis-derived preparations with low amounts of the psychoactive constituent ∆^9^-THC are not clearly addressed in the international drug control conventions. In 2019, the WHO’s ECDD recommended that preparations containing predominantly cannabidiol with trace amounts of THC should not be under international drug control, since this cannabinoid is not psychoactive and is devoid of abuse and dependence potential. Notwithstanding, the WHO’s recommendation to add a footnote to the entry for cannabis and cannabis resin in schedule I of the 1961 Convention clarifying this point was rejected by the UN’s CND in 2020 (United Nations Commission on Narcotic Drugs 2020) when it was argued that CBD is not currently controlled under the United Nations Convention on Psychoactive Substances, and therefore, no further measures would be needed (UN News 2020).

The aforementioned requirements provide a background for signatory countries to outline their regulatory frameworks regarding medical cannabis. Nevertheless, development processes have been predominantly idiosyncratic, resulting in a variety of regulatory approaches which reflect cultural, historical, and political aspects (European Monitoring Centre for Drugs and Drug Addiction 2018b; Belackova et al. 2018). Different outcomes can also be observed depending on the characteristics of these regulatory models, as well as on the local context (Seddon and Floodgate 2020; European Monitoring Centre for Drugs and Drug Addiction 2018b; Belackova et al. 2018).

**Fig. 1:**
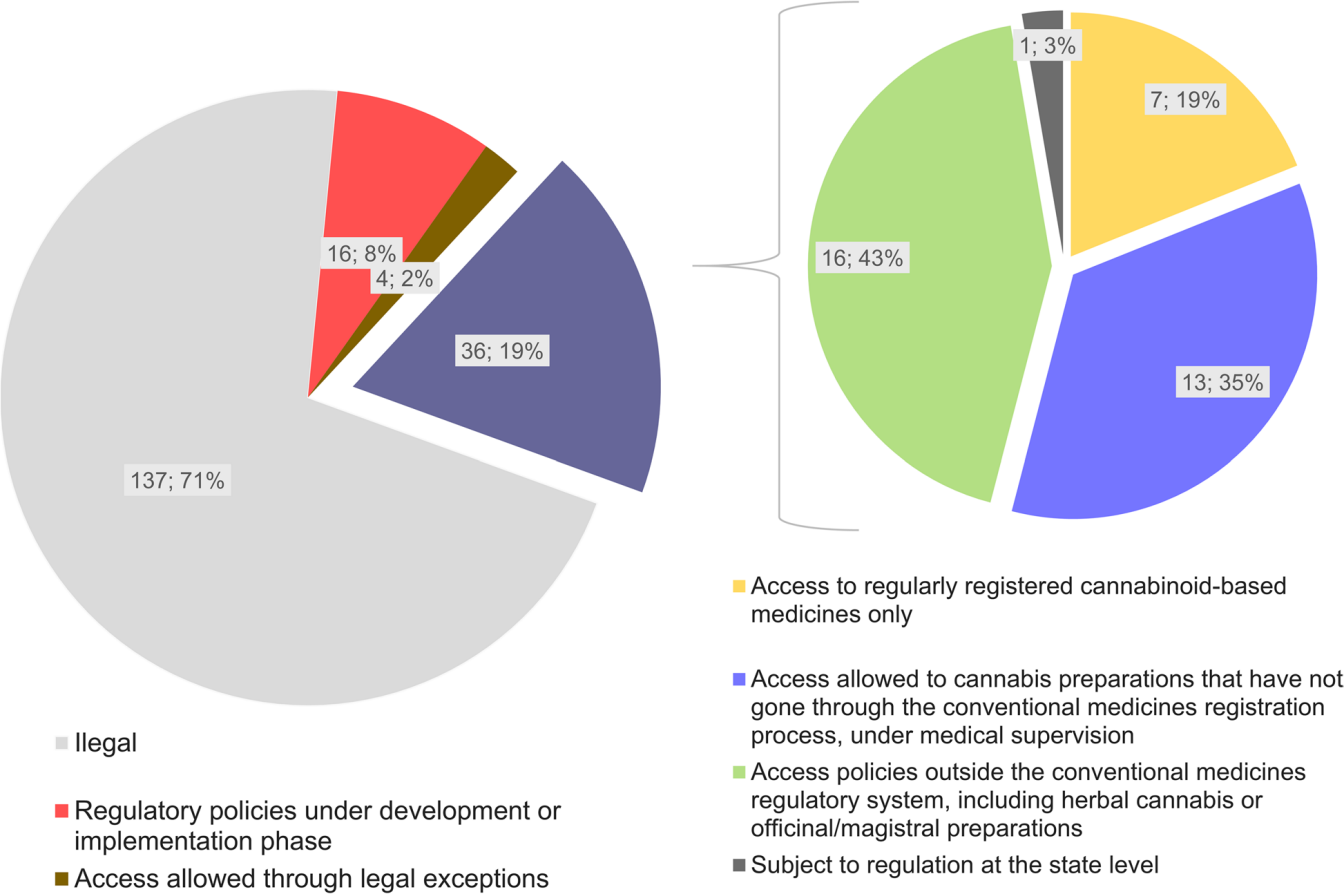
Regulatory models regarding access to medical cannabis around the world and their classification according to the types of available cannabis-based pharmaceutical products. The classification is based on data obtained from October 2020 to March 2021. The sources of the original information regarding the assessed models are listed in the supplementary material

**Fig. 2:**
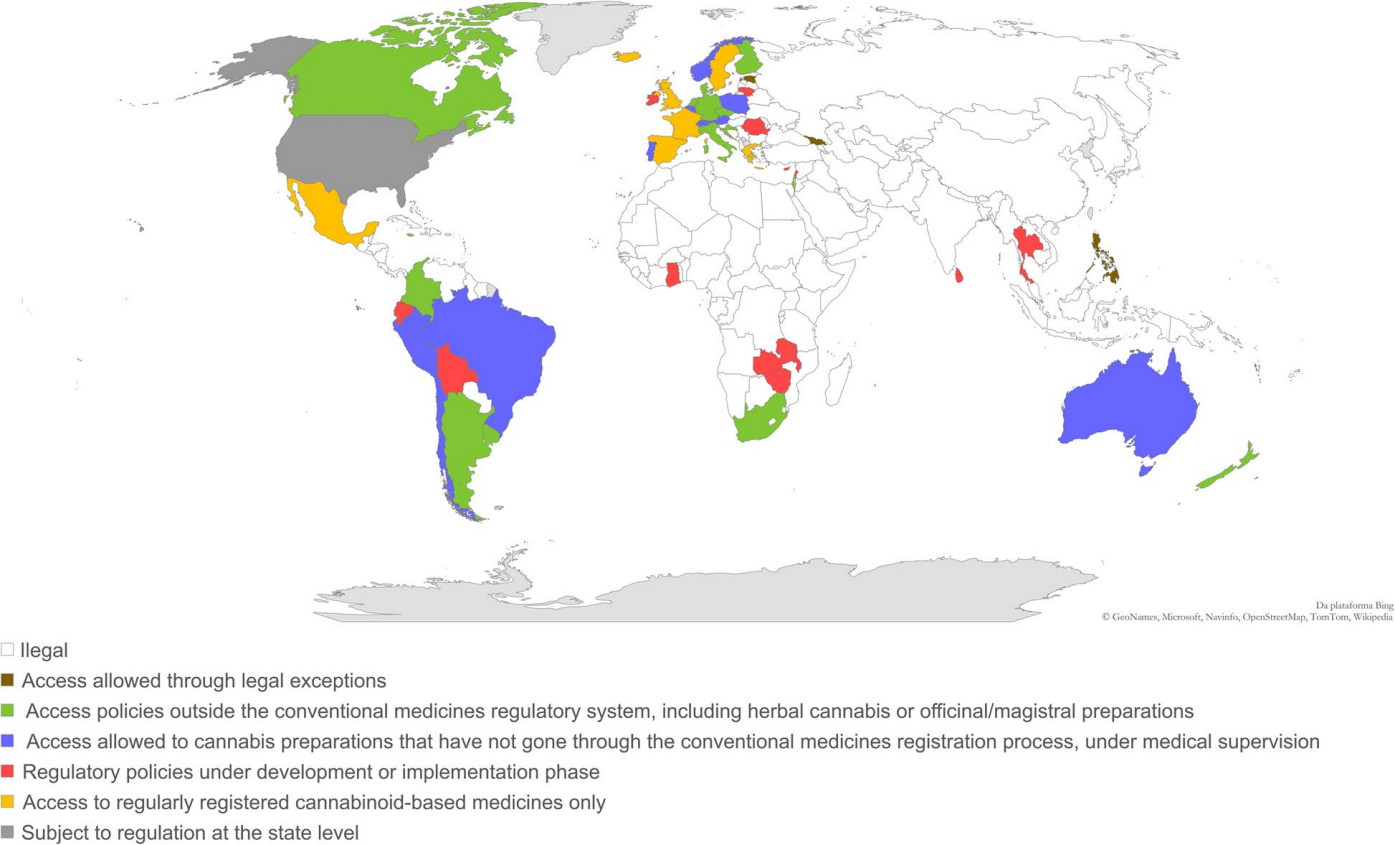
Legal status of medical cannabis and cannabis-based pharmaceutical products around the world. The classification is based on data obtained from October 2020 to March 2021. The sources of the original information regarding the assessed models are listed in the supplementary material

Information regarding regulatory models on medical cannabis already in place was found for 36 countries, which represent about 19% of the 193 sovereign states recognized by the UN. In addition, 4 countries (about 2% of UN states) allow access to medical cannabis through exceptions provided by law, and 16 countries (about 8% of UN states) currently possess regulatory frameworks under the development or implementation phase (Fig. 1). It can be noticed that most countries that currently have regulatory policies for access to medical cannabis are concentrated in the Americas and Europe (Fig. 2). Some of the elements that characterize these different regulatory approaches will be discussed below. Additional information on implemented regulatory frameworks can be found in Table S1 (Supplementary Material).

### Product types and access

Among the key aspects that may differ between different regulatory models, one can highlight the type of cannabis products that are made available for patients, along with the access mechanisms to them (European Monitoring Centre for Drugs and Drug Addiction 2018a; European Monitoring Centre for Drugs and Drug Addiction 2018b; Health Products Regulatory Authority 2017; Belackova et al. 2018). Cannabis-based medicinal products can be classified primarily between those that have undergone a regular marketing authorization process for medicines and those that have gone through more simplified authorization processes or were even exempted from specific regulatory authorizations. While for the former, it is necessary to prove their safety and efficacy through extensive non-clinical and clinical studies, in addition to demonstrating compliance with the elements of Good Manufacturing Practices (GMP), including proper quality assurance; for the latter, there is generally no guarantee that these aspects are met (Seddon and Floodgate 2020; European Monitoring Centre for Drugs and Drug Addiction 2018b).

Of the 36 regulatory models overviewed, about 19% (*n*=7) adopt strategies based exclusively or mostly on access to regularly registered products (Figs. 1 and 2). Notwithstanding the clear advantages of the conventional regulatory pathway (European Monitoring Centre for Drugs and Drug Addiction 2018b; Belackova et al. 2018), there are still few pathological conditions for which there is sufficient clinical evidence on the safety and efficacy of cannabis-based medicines (see the “Background” section). This can be attributed, at least in part, to the access difficulties arising from the strict controls imposed on cannabis, even when it comes exclusively to scientific research-related activities (Small 2015). It is also worth mentioning that the inherent variability in the chemical composition and the lack of appropriate characterization of herbal extracts are complicating factors in the process of drawing conclusions from the results of a set of different clinical trials (Food and Drug Administration 2016). This also applies to clinical trials conducted with cannabis-based medicines, which are sometimes not comparable with each other regarding the pharmaceutical formulation, dosage form, and chemical composition of the cannabis-based active pharmaceutical ingredients (API) (European Monitoring Centre for Drugs and Drug Addiction 2018b; Health Products Regulatory Authority 2017; Dinis-Oliveira 2019; Russo 2016).

Consequently, the set of cannabis-based medicines that meet the requirements for marketing authorization under the conventional pathway is still limited. As far as we know, all currently available medicines that fall within this category are characterized by containing isolated cannabinoids (or synthetic analogous) as API rather than crude *C. sativa* extracts (Health Products Regulatory Authority 2017). The most notable representatives of this type of medicines are an oral spray containing nabiximols (an association of Δ^9^-THC and CBD), indicated for the symptomatic treatment of MS-related spasticity and neuropathic pain, and a solution containing cannabidiol (100 mg/mL), indicated for the treatment of seizures related to refractory epilepsy in children, both available in several countries upon medical prescription (European Medicines Agency 2019; European Monitoring Centre for Drugs and Drug Addiction (EMCDDA), 2018; Food and Drug Administration 2018; MacCallum and Russo 2018). Dronabinol or nabilone-based medicines are also regularly registered in various countries (European Monitoring Centre for Drugs and Drug Addiction 2018b; Health Products Regulatory Authority 2017), although their use is currently less frequent. As a result, many patients’ demands end up not being fulfilled by those regularly registered medicines. Another drawback of the regularly registered cannabis medicines is their relatively high cost, which imposes additional access difficulties, especially on low-income patients (Seddon and Floodgate 2020; European Monitoring Centre for Drugs and Drug Addiction 2018b; Belackova et al. 2018).

Given the abovementioned limitations, some regulatory authorities have adopted alternative strategies to make cannabis medicinal products available (European Monitoring Centre for Drugs and Drug Addiction 2018b; Health Products Regulatory Authority 2017; Aguilar et al. 2018; Belackova et al. 2018). This is the case for about 35% (*n*=13) of the regulatory frameworks assessed in this review (Figs. 1 and 2), which provide access to cannabis-based preparations that have not gone through the conventional medicines’ approval process. In most cases, these products are supposed to be dispensed on prescription and should be used under medical supervision. As the therapeutic indications for this type of cannabis products are not defined based on clinical studies presented during the marketing authorization process, some regulatory approaches include the pre-definition of a set of eligible diagnoses (e.g., Portugal, Italy, Czech Republic), while others leave this decision the responsibility of the prescribing professionals (e.g., New Zealand). There are also situations in which eligible diagnoses are limited to “potentially life-threatening” conditions, but not exhaustively identified in legislation (e.g., Switzerland) (European Monitoring Centre for Drugs and Drug Addiction 2018b; Aguilar et al. 2018; Belackova et al. 2018; Abuhasira et al. 2018) (Table S1, Supplementary Material).

**Fig. 3:**
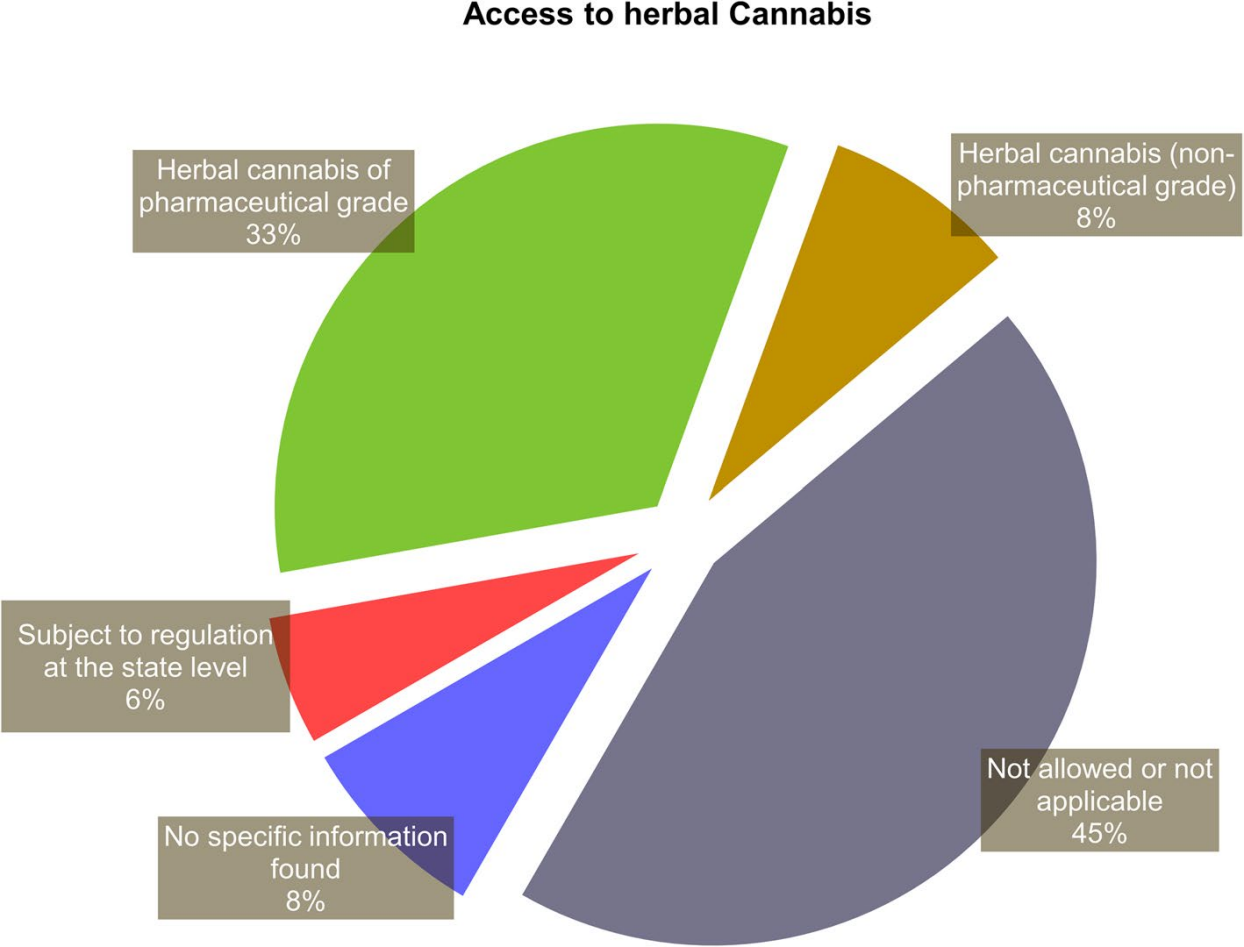
Classification of the assessed regulatory models with regard to the possibility of access to herbal cannabis. The classification is based on data obtained from October 2020 to March 2021. The sources of the original information regarding the assessed models are listed in the supplementary material

**Fig. 4:**
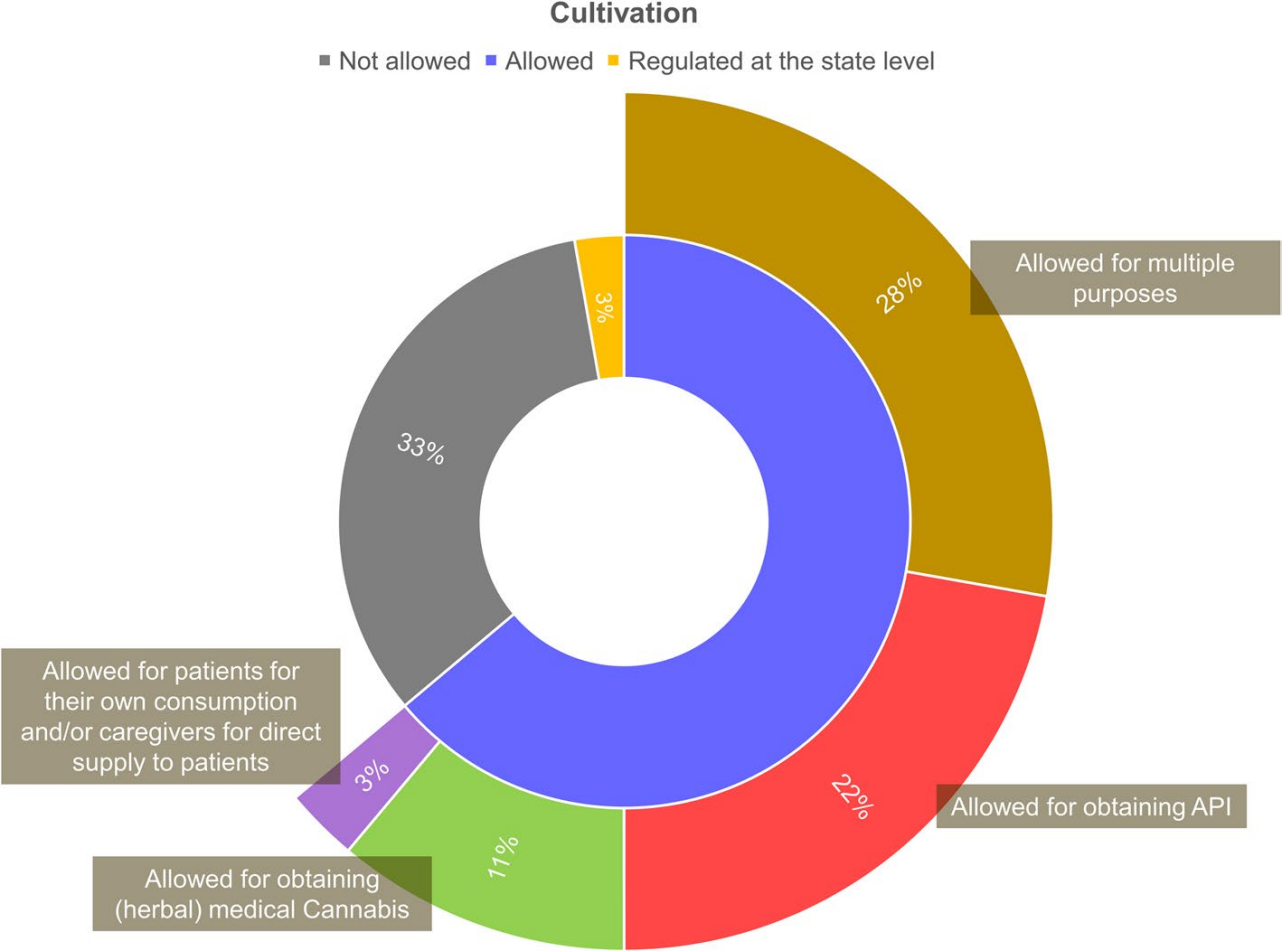
Classification of the assessed regulatory models with regard to the possibility of medical cannabis cultivation and the purposes of that activity. The classification is based on data obtained from October 2020 to March 2021. API, active pharmaceutical ingredient. The sources of the original information regarding the assessed models are listed in the supplementary material

In addition to the aforementioned approaches, there are others that include access policies to cannabis products outside the medicines regulatory system (Seddon and Floodgate 2020; European Monitoring Centre for Drugs and Drug Addiction 2018b; Health Products Regulatory Authority 2017; Aguilar et al. 2018; Belackova et al. 2018; Abuhasira et al. 2018; Klieger et al. 2017). This was the case for about 43% (*n*=16) of the assessed national-level regulatory models, as well as for most of the state-level regulations in the USA (Figs. 1 and 2). Most of those schemes include access to herbal cannabis, with varying levels of quality requirements, ranging from pharmaceutical-grade medicinal cannabis (e.g., Dutch model) to self-cultivated or non-controlled plant materials (Figs. 3 and 4) (see also “Cultivation and supply regulation”).

One important drawback of those more permissive approaches is the lack of confidence among physicians to recommend the use of medical cannabis without the proper support of clinical evidence (Seddon and Floodgate 2020; European Monitoring Centre for Drugs and Drug Addiction 2018b; Health Products Regulatory Authority 2017; Schlag 2020). Indeed, the widespread use of unregistered cannabis products that have not undergone clinical trials is far from ideal, especially since *C. sativa* is a highly variable plant species with a complex phytochemical profile, including secondary metabolites with a relatively narrow therapeutic margin (e.g.: Δ^9^-THC) (Dinis-Oliveira 2019; Crippa et al. 2016; Koltai et al. 2019).

Moreover, unregistered medical cannabis preparations are often obtained in breach of Good Agricultural Practices (GAP) or Good Manufacturing Practices (GMP), which raises serious concerns regarding quality issues, among which heterogeneity of cannabinoid concentration stands out (World Health Organization 2014; Health Products Regulatory Authority 2017; Dinis-Oliveira 2019). As a result, batch-to-batch consistency of the safety-efficacy profile may not be guaranteed, which compromises the promotion of rational use of medical cannabis (Health Products Regulatory Authority 2017; Dinis-Oliveira 2019).

It is also worth mentioning that access schemes outside the medicines regulatory system may discourage the conduct of appropriate clinical trials, in addition to making it more difficult to systematically assess the benefits and risks associated with the use of medical cannabis (European Monitoring Centre for Drugs and Drug Addiction 2018b; Health Products Regulatory Authority 2017).

In order to mitigate the possible negative impacts of using less regulated cannabis products, some access policies limit their indication to patients with debilitating or life-threatening conditions who are refractory to treatment with conventional medicines (European Monitoring Centre for Drugs and Drug Addiction 2018b; Krcevski-Skvarc et al. 2018) (Table S1, Supplementary Material). For those patients, the possibility of improving life quality outweighs the possible risks. This understanding is also at the basis of compassionate use schemes, which allow access to medicines under clinical investigation or regulatory approval phase (Seddon and Floodgate 2020; European Monitoring Centre for Drugs and Drug Addiction 2018b; Health Products Regulatory Authority 2017; Aguilar et al. 2018; Belackova et al. 2018; Abuhasira et al. 2018). Some of the national regulatory models overviewed (*n*=4) were primarily based on exceptional or compassionate use programs, which included strategies such as individual import authorization and exemption from criminal prosecution. Although restricted, these access policies usually serve as a starting point for more elaborate regulatory frameworks (Health Products Regulatory Authority 2017).

Patient authorization mechanisms may also vary from country to country. Most accessed schemes are based on conventional medical prescriptions (Table S1, Supplementary Material). Schemes based on “practitioner recommendation” are mainly observed in the US state-level regulatory models (Health Products Regulatory Authority 2017; Klieger et al. 2017). Administrative approvals such as individual patient registration are also required in some cases, especially for regulatory approaches based on access to unregistered cannabis-based preparations (European Monitoring Centre for Drugs and Drug Addiction 2018b; Aguilar et al. 2018; Belackova et al. 2018).

### Cultivation and supply regulation

The assessed regulatory models also differed concerning medical cannabis supply. Four distinguishing aspects can be highlighted on this topic: (I) cannabis cultivation (whether it is allowed or not and for what purposes); (II) the extent of domestic supply (whether pharmaceutical preparations are imported or manufactured domestically and, in this case, whether the API is imported or not); (III) the production licensing (whether growing and manufacturing activities are centralized by government agencies or undertook by licensed private companies); and (IV) distribution mechanisms (whether this activity is centralized or not).

Most of the implemented regulatory models overviewed in this study (about 67%) provide for cannabis cultivation to some extent. In certain countries, cultivation is limited to API obtention to be supplied to the pharmaceutical industry, under controlled conditions (e.g., UK, Greece, Mexico), while others have models that allow cultivation for obtaining herbal medical cannabis to be further dispensed to patients (e.g., Netherlands, Italy) (European Monitoring Centre for Drugs and Drug Addiction 2018b; Aguilar et al. 2018; Belackova et al. 2018). Besides, there are those with more comprehensive models, which provide for the possibility of cultivation for different purposes (e.g., Uruguay, Canada, New Zealand) (European Monitoring Centre for Drugs and Drug Addiction 2018b; Aguilar et al. 2018; Belackova et al. 2018; Rychert et al. 2019; Santé Canada 2019) (Table S1, Supplementary Material).

Regardless of the aforementioned distinctions, countries that authorize Cannabis cultivation adopt a series of control measures, including the granting of specific licenses by the government, according to the scale and purpose of cultivation (Health Products Regulatory Authority 2017; Belackova et al. 2018). Certification on GAP is also required in some countries (e.g., Italy, Netherlands, Canada, New Zealand, Israel) so as to control batch-to-batch variation and limit microbiological and chemical contamination (e.g., pesticides, heavy metals) of the herbal material (European Monitoring Centre for Drugs and Drug Addiction 2018b; Aguilar et al. 2018; Belackova et al. 2018; Rychert et al. 2019; Santé Canada 2019). Similarly, the manufacturing of cannabis-based pharmaceutical products on an industrial scale must also comply with GMP requirements and are generally conditional on the granting of specific licenses (European Monitoring Centre for Drugs and Drug Addiction 2018b; Health Products Regulatory Authority 2017; Aguilar et al. 2018; Belackova et al. 2018). Additional requirements concerning facility security, such as access controls, crop integrity assurance, and disposal or destruction of cannabis remains, may also apply in some cases (e.g., in New Zealand, South Africa, and some of the US state-level regulations) (Seddon and Floodgate 2020; European Monitoring Centre for Drugs and Drug Addiction 2018b; Klieger et al. 2017; South African Health Products Regulatory Authority 2019; Medicinal Cannabis Agency 2020).

The state can assume different roles regarding the cannabis supply chain regulation, ranging from the simple monitoring of the activities carried out by licensed companies (e.g., most state-level regulations in the USA) to schemes in which a closed production chain is established, with a government agency centralizing the acquisition and distribution of all medicinal cannabis grown in the national territory (e.g., Netherlands) (European Monitoring Centre for Drugs and Drug Addiction 2018b; Belackova et al. 2018; Klieger et al. 2017; Bureau voor Medicinale Cannabis 2021). The most centralized models of supply chain regulation are often associated with better control of possible risks to patients, including those related to quality issues and deviations from intended use, although they generally offer less flexibility in terms of access options (Belackova et al. 2018). The costs involved and the structure available to government agencies are also factors to be considered when defining the best regulatory approach. Most of the regulatory models assessed in this study adopted non-centralized approaches, with a government body/agency responsible for authorizing and monitoring the supply chain activities (Table S1, Supplementary Material).

Some regulatory models also provide for self-cultivation by patients or cultivation by caregivers or associations for direct supply to patients, either alone or in parallel with large-scale cultivation (European Monitoring Centre for Drugs and Drug Addiction 2018b; Aguilar et al. 2018; Belackova et al. 2018; Abuhasira et al. 2018; Klieger et al. 2017). Self-cultivation is often conditioned to the obtaining of previous individual authorization from regulatory authorities, which, in most cases, is dependent on medical recommendation. Several regulatory approaches establish clear limits on the number of cannabis plants or the amounts of herbal cannabis the patient may keep for personal consumption. Such limits, however, vary significantly from country to country (Table S1, Supplementary Material). It is not uncommon for patients or associations that practice the authorized cultivation of medicinal cannabis to prepare extracts and other types of processed materials so as to allow the use by routes other than inhalation (Cáceres Guido et al. 2020).

Although self-cultivation is sometimes advocated as a less costly access alternative, this practice raises several concerns regarding the risks to which patients are exposed when using such “homemade” products (Dinis-Oliveira 2019). The lack of standardization and appropriate controls inherent to this type of activity results in the obtaining of heterogeneous materials with unpredictable toxicological and pharmacological effects (Dinis-Oliveira 2019; Cáceres Guido et al. 2020). In fact, this would be an additional variability factor to deal with in therapeutic practice, adding to the inherent variability of the species, the complexity of its phytochemical profile, and the variability of individual patients’ responses (Dinis-Oliveira 2019).

### Technical requirements

The technical requirements applied to cannabis-based products are another relevant distinguishing aspect of the regulatory models. The way criteria related to production, quality control, safety/efficacy assessment, labeling, and packaging are addressed directly depends on the product categories in question. For cannabis-based medicines submitted to a regular marketing authorization process, technical criteria are generally well defined within the scope of the medicine regulatory system. In turn, the technical criteria imposed on unregistered cannabis products may vary depending on the regulatory framework (Seddon and Floodgate 2020; European Monitoring Centre for Drugs and Drug Addiction 2018b; Russo 2016; Aguilar et al. 2018; Belackova et al. 2018). For instance, some regulatory schemes require that unregistered products are manufactured according to GMP requirements and that their relevant quality attributes are monitored so that batch-to-batch consistency can be ensured (e.g., Canada, New Zealand). Others include non-industrialized products, such as officinal and magistral preparations, which may lack appropriate quality control and standardization (MacCallum and Russo 2018) (Table S1, Supplementary Material).

For regulatory models that provide access to unregistered products, the need to prevent risks associated with exposing patients to low-quality, adulterated, or contaminated products is a major concern. In this regard, the search for the definition of appropriate parameters of identity, purity, and cannabinoid content applied to cannabis-based API and pharmaceutical products is worth noting (World Health Organization 2014; Sarma et al. 2020).

Among the critical quality issues to be considered regarding herbal cannabis, one can highlight the need to characterize and standardize the cannabinoid profile and to guarantee that the plant material is free from microbiological (e.g., bacteria and fungi) and chemical contamination (e.g., heavy metals and pesticides) (World Health Organization 2014; European Monitoring Centre for Drugs and Drug Addiction 2018b; Sarma et al. 2020). On this topic, the known susceptibility of herbal cannabis to contamination by fungi of the species *Aspergillus fumigatus* L. also raises concerns about the possibility of the presence of mycotoxins (World Health Organization 2014; Sarma et al. 2020). The limits established for the aforementioned impurities are most often based on the general acceptance criteria defined in official compendiums applicable to API of botanical origin, although, in some cases, the establishment of specific criteria, based on risk analysis and the precautionary principle, considering factors such as the sample’s particularities, the target population, and the intended forms of use, may apply (Sarma et al. 2020; Upton et al. 2013). Monitoring the stability of cannabis-based API and pharmaceutical products through the assessment of relevant quality attributes is also a challenge (European Monitoring Centre for Drugs and Drug Addiction 2018b).

In this context, stand out the development of some quality monographs on cannabis inflorescences, including the German Pharmacopoeia monograph (Deutsches Arzneibuch n.d.) and the quality specifications, officially adopted by the government agencies responsible for medical cannabis in the Netherlands (Bureau voor Medicinale Cannabis 2014), Denmark (Sundheds-og Ældreministeriet 2019), and New Zealand (New Zealand 2019). The monograph published by the American Herbal Pharmacopoeia (AHP) (Upton et al. 2013) is also a commonly used guideline, mainly within the USA. It is also worth mentioning that initiatives from organizations such as the Association of Official Analytical Chemists (AOAC) International and the American Society for Testing and Materials (ASTM) have engaged different stakeholders in the development of independent quality standards and guidelines, which may help to construct a set of appropriate analytical methods and quality specifications for medical cannabis (Sarma et al. 2020).

## Medical cannabis in Brazil: status and perspectives

In Brazil, the species *C. sativa* is included in List “E” of *Portaria* SVS/MS N° 344/1998, which represents a list of banned plants that can originate narcotic and/or psychotropic substances (BRASIL 1998). In 2006, the Law N° 11.343/2006 provided for the possibility of government authorization of the species cultivation, exclusively for medicinal or scientific purposes, under the state’s surveillance (Brasil 2006), which is coherent with what is already foreseen in the international treaties in force. Nonetheless, the regulation of this activity remained pending, hindering its execution from a practical standpoint.

Since 2015, there have been some notable advances toward allowing the medicinal use of the species in the country. With the publication of ANVISA RDC N° 03/2015, cannabidiol was moved from List “F” (Banned substances in Brazil) to list “C1” (list of other substances subject to special control) of the *Portaria* N° 344/1998 (Agência Nacional de Vigilância Sanitária 2015a). In the same year, the import of CBD-containing products for medical use was simplified with the publication of ANVISA RDC N° 17/2015 (Agência Nacional de Vigilância Sanitária 2015b). This was mostly motivated by reports of CBD effectiveness in the treatment of refractory epilepsy in children, resulting in a remarkable demand by patients’ families for this therapeutic alternative (Oliveira 2017).

Parallel to this, discussions about the viability of the medicinal use of the species gained ground in the national congress. In this context, some bills were presented, among them the PL 399/2015 (Brasil 2015), which aimed at amending the current legislation to allow the marketing of medicines that contain extracts, substrates, or parts of the *C. sativa* plant in their formulation.

Later in 2016, ANVISA RDC N° 130/2016 authorized the prescription, exclusively by doctors, of cannabis-based medicines intended for the human use provided that they received marketing authorization from ANVISA (Agência Nacional de Vigilância Sanitária 2016). In 2017, Mevatyl® (Nabiximols) became the first cannabis-based medicine to receive marketing authorization in Brazil, with the therapeutic indication for MS-related spasticity (Agência Nacional de Vigiância Sanitária 2017).

In 2019, the regulatory category of “cannabis products” was created by ANVISA, which made it possible to obtain a simplified authorization for cannabis-based products to be marketed in Brazil, valid for up to 5 years (Agência Nacional de Vigilância Sanitária 2019). According to the ANVISA RDC N° 327/2019, a set of minimum regulatory requirements applies to those products, including the certification of the production site on GMP and the evaluation of basic quality parameters, including the fulfillment of criteria established in pharmacopeial monographs, if available. However, authorization applicants were exempted from the presentation of safety and efficacy proofs. This regulatory approach was mainly driven by the increased demand for the availability of these products in the domestic market.

The aforementioned statute further determines that access to cannabis products is subject to prescription by a qualified health professional, who is responsible for defining the indications and appropriate dosage, according to the patients’ clinical conditions. Moreover, dispensing of cannabis products is controlled, being conditioned to the presentation of special prescription notification “A” or “B”, depending on the declared Δ^9^-THC content. Prescription of cannabis products with Δ^9^-THC content greater than 0.2% is limited to terminally ill patients or those who are refractory to available therapeutic options (Agência Nacional de Vigilância Sanitária 2019).

Brazilian residents can also get access to cannabis-based pharmaceutical products through individual import authorization in accordance with ANVISA RDC N° 335/2020 (Agência Nacional de Vigiância Sanitária 2020). However, many of the imported products have not been subject to regulatory approval as medicines in countries of origin; therefore, relevant parameters regarding their quality, safety, and efficacy may have been neglected. Furthermore, the costs of acquiring these products via importation are generally very high when considering the socioeconomic reality of most Brazilians (de Oliveira et al. 2020). Consequently, it is common for interested patients to apply to the courts for these products to be paid for by the public health system, which is often granted (de Oliveira et al. 2020). However, in view of the overall low quality of most of those products, there is no evidence of a favorable cost-benefit ratio in this situation.

Faced with the limited availability of affordable cannabis medicinal products, many patients end up resorting to lower-cost access alternatives such as self-cultivation, which is not regulated, or clandestine distribution networks. There are also non-profit associations of patients that have obtained exemptional legal authorizations for cannabis cultivation and preparation of cannabis extracts for medicinal purposes (de Oliveira et al. 2020). Indeed, it is not uncommon for patients who have experimented with using imported non-pharmaceutical grade products to hypothesize that self-cultivation would be a lower-cost strategy that would provide equivalent results (de Oliveira et al. 2020). This reasoning, however, establishes a false dichotomy between the two options (i.e., high-cost and dubious quality imported products versus non-pharmaceutical grade “homemade” extracts), while neglecting other more appropriate access possibilities. Other problematic aspects commonly identified in the discourses of self-cultivation proponents, which somehow reflect certain ideas widespread in Brazilian popular culture, are the naturalistic fallacy, arguing that products of “natural” origin would be safe, and the over-emphasis on the value of personal experiences and alleged “traditional uses” as evidence of efficacy (de Oliveira et al. 2020; Bacchi 2020). In addition, there is a lack of understanding among the lay population that cannabis-based products with therapeutic claims are complex in terms of composition, preparation process, and pharmacological effects and, therefore, should be treated (and controlled) as medicines. Those misperceptions often favor the non-appropriate use of “homemade” cannabis-based preparations by patients, especially by vulnerable groups (e.g., pediatrics), thus putting them at risk due to the lack of adequate standardization and control of relevant quality attributes (see “Cultivation and supply regulation”) (Dinis-Oliveira 2019; Cáceres Guido et al. 2020).

In view of the above, it is noticeable that, despite the approaches adopted in recent years to expand the availability of cannabis-based products in Brazil, a large portion of the interested patients still have limited access to quality pharmaceutical products. Moreover, there are still pending issues for the following years, including the regulation of the species’ cultivation in the national territory and its access for research purposes.

PL 399/2015 (Brasil 2015), a bill currently under discussion in the Brazilian National Congress, provides for the regulatory framework of cannabis in Brazil, addressing some of the aforementioned topics. The current text of this legislative proposal provides for *C. sativa* cultivation within the national territory by authorized legal entities for medical and scientific purposes. If approved, this may favor the access to cannabis-based API by pharmaceutical industries and research institutes. The document also foresees the possibility of cannabis cultivation by authorized non-profit patients’ associations and “*Farmácias Vivas*” (pharmacies dedicated to the cultivation of medicinal plants and formulation of magistral or officinal herbal medicines within the Brazilian public health system), providing certain criteria to ensure that the quality of the obtained products is minimally controlled (Brasil 2015). However, the effectiveness of these measures will depend on how they are implemented and the enforcement mechanisms established. Regarding the patient’s access mechanisms, the bill in question maintains similar criteria compared to those currently in place under RDC 327/2019.

One of the challenges of medical cannabis regulation is to achieve a balance between guaranteeing patients access to the products they need and controlling the risks inherent to their use (Belackova et al. 2018). In an ideal scenario, the development of regularly registered cannabis-based medicines should be prioritized, for which the relevant aspects of safety, efficacy, and quality are properly investigated (Health Products Regulatory Authority 2017). This favors the construction of a solid basis for the rational use of these products. On the other hand, higher regulatory standards generally imply higher costs, which can hinder patient access, unless appropriate strategies are adopted by the government (Belackova et al. 2018). The establishment of price control policies for cannabis-based medicines is necessary to prevent abusive practices from being adopted and patients from being driven to resort to the illegal cannabis market (or self-cultivation) instead (Seddon and Floodgate 2020; Aguilar et al. 2018; Schlag 2020). Moreover, in the Brazilian scenario, the existence of a universal public health system (*Sistema Único de Saúde,* SUS) can favor the development of successful access mechanisms. Indeed, PL 399/2015 provides the possibility that cannabis-based products and medicines may be included within the scope of this system (Brasil 2015). However, it is worth mentioning that any costs imposed on the public health system should be outweighed by the proven benefits of the interventions (Bacchi 2020). In this context, it would be important that the cannabis-based products subsidized by the Brazilian public health system should have their efficacy and safety duly based on clinical evidence. Notwithstanding, this condition is neglected in the abovementioned bill.

The definition of relevant quality standards for cannabis-based API and products is also of ultimate importance. In this context, the development of pharmacopeial monographs for *C. sativa* plant material and extracts may contribute to the standardization of quality specifications, especially regarding the sample’s identity, purity, chemical profile, and cannabinoid content, thus reducing the possibility of adulteration and preventing the use of contaminated materials (Giancaspro et al. 2016; Sarma et al. 2020).

The promotion of an environment that encourages research and innovation is also an important factor so that well-designed products with favorable safety-efficacy profiles are made available to the population (Health Products Regulatory Authority 2017; Dinis-Oliveira 2019). The definition of clear guidelines for the construction of the set of safety and efficacy proofs required for registration or marketing authorization of cannabis-based medicines is also an important aspect to be considered. As the safety-efficacy profile depends directly on the characteristics of the herbal API and the product itself, as well as on the use regimens and patient’s clinical condition (Health Products Regulatory Authority 2017), specific studies should be carried out with the medicine subject to regulatory approval in order to allow drawing conclusions concerning its suitability for the therapeutic claims in question.

Furthermore, monitoring the adverse effects of cannabis-based products available on the market, through an adequate pharmacovigilance system, is of great importance so as to ensure the risk-benefit ratio remains favorable over time for patients who use them (Health Products Regulatory Authority 2017; Schlag 2020). The collection of safety data also allows the development of an evidence base that may further favor the regulatory framework improvement (Schlag 2020).

Experiences from other countries also indicate the need for continuous improvement of the regulatory framework over time, in order to keep up with the evolution of scientific knowledge and patients’ demands. In this process, maintaining an open dialog between different stakeholders, including members of the scientific community, regulators, prescribing professionals, and patients can favor meeting the demands of society while maintaining the scientific rigor necessary to the subject (Health Products Regulatory Authority 2017; Aguilar et al. 2018; Schlag 2020). It is also worth mentioning the need to provide an appropriate education for patients, in such a way that their concerns are addressed, so that they can understand the risks and benefits of therapy with cannabis-based products and dispel false and skewed views about them (Health Products Regulatory Authority 2017; Schlag 2020).

## Conclusion

The debate over the medical use of *C. sativa* has often been permeated by a Manichaean logic. On the one hand, the stigma created over many decades on the plant species has favored that several stakeholders advocate strictly prohibitionist policies, overestimating risks and failing to recognize the already proven benefits arising from its therapeutic use in certain conditions (Seddon and Floodgate 2020; Aguilar et al. 2018). On the other hand, there are those who support indulgent access policies, based mainly on the popularly widespread misperception that *C. sativa* and its constituents would be effective for countless clinical conditions, and that, because of their “natural” origin, there would be no safety concerns related to their use (Health Products Regulatory Authority 2017; Dinis-Oliveira 2019). Both of those “black-or-white” perspectives carry biases that can make it difficult to make rational and science-based decisions during the development of regulatory frameworks on this subject. In view of the “post-truth era” conjuncture, understanding the permeability of regulatory policies to a society’s values, beliefs, and prejudices is essential in order to avoid adopting practices that could have a negative impact on public health instead of protecting it and reducing harms (Alves 2020).

As well as other medicines, medical cannabis and its derivatives should have their use based on evidence of quality, safety, and efficacy (Health Products Regulatory Authority 2017; Government of Australia 2016). Nevertheless, the tendency to avoid investigating sensitive or controversial subjects, along with the difficulties imposed by international prohibitionist policies, has hampered scientific research on the therapeutic and pharmacological properties of the species, resulting in a knowledge gap that remains nowadays (Small 2015). As a consequence, the scientific data that would be required for the regularization of cannabis-based medicines through conventional regulatory pathways are sometimes insufficient (Health Products Regulatory Authority 2017). In this scenario, public perception often identifies regulatory requirements as “bureaucratic” barriers to accessing medical cannabis products, while the greatest obstacle to be overcome is often the scarcity of scientific evidence (Health Products Regulatory Authority 2017). Therefore, the importance of developing regulatory frameworks that provide mechanisms to encourage scientific research, both by facilitating access and providing financial resources, is emphasized so as to favor the construction of a knowledge basis on which the safe and effective use of the species can be supported (Health Products Regulatory Authority 2017; Cáceres Guido et al. 2020).

In addition, it is important to keep in mind the purposes of the medical cannabis regulation, with an emphasis on reducing risks to patients, as well as to develop mechanisms for clear communication with interested parties so they can understand both strengths and limitations of the regulatory choices. At the same time, regulators and policymakers should be able to recognize and be sensitive to the demands of the population, seeking to include them in the decision-making process (Health Products Regulatory Authority 2017). Finally, the construction of regulatory models should be understood as a continuous process, and there must be spaces for constant improvement according to the observed outcomes and the evolution of scientific knowledge.

## Supplementary Information

**Additional file 1: Table S1.** Regulatory models

## Data Availability

The dataset generated and analyzed during the current study is available in the supplementary material.

## Abbreviations

AHP: American Herbal Pharmacopoeia
ANVISA: *Agência Nacional de Vigilância Sanitária* (Brazil)
API: Active pharmaceutical ingredient
CBD: Cannabidiol
CND: UN’s Commission on Narcotic Drugs
ECDD: WHO’s Expert Committee on Drug Dependence
GAP: Good agricultural practices
GMP: Good manufacturing practices
INCB: International Narcotics Control Board
Δ^9^-THC: Tetrahydrocannabinol
UN: United Nations
WHO: World Health Organization
